# Effect of smoking and refrigeration on lipid oxidation of *Clupea harengus*: A fish commonly consumed in Cameroon

**DOI:** 10.1002/fsn3.575

**Published:** 2017-12-21

**Authors:** Noel Tenyang, Bernard Tiencheu, Hilaire Macaire Womeni

**Affiliations:** ^1^ Department of Biological Sciences Faculty of Science University of Maroua Maroua Cameroon; ^2^ Department of Biochemistry Faculty of Science University of Dschang Dschang Cameroon; ^3^ Department of Biochemistry Faculty of Science University of Buea Buea Cameroon

**Keywords:** FTIR spectroscopy, herring, lipid oxidation, refrigeration, smoking

## Abstract

Changes in lipids of herring (*Clupea harengus*) muscle during different smoking methods and 9 days of refrigeration at 4°C were investigated. The proximate analysis of raw herring revealed that the total lipid, the total protein content and the crude ash were 10.20%, 69.43%, and 19.42%, respectively. The results also indicated that during all processing free fatty acids (FFAs) and peroxide value (PV) increased, whereas iodine value (IV) and total triglycerides decreased. The change were more significantly (*p* < .05) during unbleached + hot smoking and refrigeration at more than 6 days. All these modification decrease the nutritional value of herring. Bleaching combined to smoking and refrigeration at <6 days had more desire effect on lipid oxidation of herring compared to other treatments. The fish obtained in these conditions are more suitable for feeding heath nutrition.

## 1 INTRODUCTION

The number of undernourished people worldwide has been estimated to have increased to 963 million compared to 923 million in 2007 (FAO, 2008). This estimation confirms the need for immediate attention considering, the ongoing financial, and economic crisis. In the recent years, fish has been reported as a good source of protein used to correct protein deficiency in human diets in tropic region (Akinwumi, 2011). Fish provide animal proteins that consist of all the essential amino acids in relatively high concentrations, low in cholesterol and saturated fatty acid, and also rich in essential polyunsaturated fatty acid, minerals, and vitamins. Diverse benefits for human heath, due to the consumption of polyunsaturated fatty acids (PUFA) recently has been widely recognized (Gladyshev, Sushchik, Gubanenko, Demirchieva, & Kalachova, 2007).

The beneficial effects of omega‐3 fatty acids especially eicosapentaenoic acid (EPA) and docosahexanoic acid (DHA) are mainly seen with fatal cardiovascular disease. More specifically, the most pronounced effects of omega‐3 fatty acids are shown on sudden death. EPA and DHA acids plays a preventive role for a number of diseases in humans such as inflammation, cancers. EPA and DHA also are involving in lowering blood pressure and cholesterol levels, which is essential for cardiovascular health (Schram et al., 2007). Aquatic systems are the main sources of the two essential PUFAs in the biosphere, and humans obtain these fatty acids through fish and, marine and freshwater products (Kolakowska, Zienkowicz, Domiszewski, & Bienkiewicz, 2006). The fatty fish such as herring, catfish are very popular and important for domestic consumption because of the good nutritional properties of polyunsaturated fatty acids (Tenyang et al., 2013). Alvarez, Medina, Pegro, and Aubourg (2009) reported that the concentration of n‐3 PUFA and total lipid varied between the types of fish, climate, nutrition, origin, and part of the fish that meat comes from.

After catching, fish meat is probably the most susceptible to alteration mainly because of its high concentration of unsaturated fatty acids and its shelf life is limited by the enzymatic and microbial activity (Cho, Endo, Fujimoto, & Kaneda, 1989). Since many years, smoking, refrigeration, and frozen were the main methods used for the preservation of food. Not only smoking extends the shelf life of food via the effect of dehydratation and antioxidant action of the smoke compound, which has the effect on microbial activity, smoking also gives the special color and flavor to the food. During smoking treatment, changes on the texture of product have also been observing (Goulas & Kontminas, 2005). Frozen and refrigeration are the cold treatment which used the lowing temperature to preserve the quality of food by limiting degradation due to the lowest activities of microorganisms (Alcicek & Atar, 2010). Smoking and refrigeration used commonly to conserve food retain the nutritional properties of food (Badii & Howell, 2002).

Fatty fish is a rich source of polyunsaturated fatty acids and the degradation of theses PUFAs by hydrolytic degradation and autooxidation during processing are easily leads to the formation of free fatty acids, peroxides compounds, and the formation of volatiles molecules, which are involved in the rancidity of fish oils (Pasoz, Gallardo, Torres, & Medina, 2005). During processing, oxygen comes into contact with fish oil or fat and then the fats or oils turn rancid, resulting in unpleasant flavors. The oxidation process of PUFAs is catalyzed by heat, light, or enzymes (Bremner, 2002).

Despite the various studies focusing on the effect of cooking, smoking, and freezing on the lipid oxidation of fish as far as we know (Andrzej, Justyna, & Piotr, 2015; Bognar, 1998; Tenyang et al., 2013), no data has been reported regarding the effect of smoking and refrigeration on lipid oxidation of herring fish consumed in Cameroon. Moreover, it is not well‐known in which degree these treatments affect the quality of herring fish oil. Thus, the aim of this work was to investigate the effect of smoking and refrigeration on lipid oxidation of herring consumed in Cameroon.

## 2 MATERIAL AND METHODS

### 2.1 Fish samples collection

Herring (*Clupea harengus)* chosen for this work is the commonly specie consumed in Cameroon. They were collected during April month. Eighteen samples of herring were purchased from the main representative fish market in Douala (Youpoue). The Youpoue market located along the Wouri river is renowned for its diversity of fish. This is a great center for supply of fresh fish, also a transit center of smoked fish in the direction of other markets in Cameroon. The mean weight and length of the fish purchased were 200 ± 0.5 g and 25 ± 2.25 cm, respectively. These fish originated from the marine coast in Cameroon. After being purchased, they were transferred on the laboratory in ice containing boxes within 3 hr.

### 2.2 Chemicals

All the chemicals, solvents used in this work were of analytical grade and obtained from Sigma–Aldrich (St. Louis, USA), SD‐fine Chemicals limited (Ahmedabad, India), and Courtage Analyses Services (Mont Saint‐Aignan, France). All reagents used were of laboratory grade.

### 2.3 Smoking and refrigeration methods

#### 2.3.1 Smoking procedures

The herring used for smoking method were in two types: bleached and unbleached herring. The raw herring are spread out on smoking trays after washing without salting. The trays are then sacked on smoking oven fired with hard wood, and marked at temperature greater than 70°C. The smoking procedure takes a period of 7 hr to obtain a dry smoked fish. During smoking, fish were turned at intervals. A sample of raw herring were homogenized and used as an untreated control sample.

#### 2.3.2 Refrigeration procedures

For refrigeration, whole herring were immediately washed, package in polyethylene bags, and stored at 4°C in refrigerator for 9 days. During refrigeration, two samples of fish were taken as the composite sample at 0, 3, 6, and 9 days for analyses. The fish samples after refrigeration were washed and filleted.

#### 2.3.3 Lipid extraction

After the different smoking and refrigeration treatments of herring, lipids were extracted according to Bligh and Dyer method (1959) using a combination of chloroform, methanol, and distilled water (1:2:1). After extraction, the extracts were stored in dark glass bottles at a temperature of −4°C for further analysis.

### 2.4 Analytical methods

#### 2.4.1 Proximate analysis of raw sample

Moisture, fat, ash, and protein in the raw sample were determined in triplicate using standard analytical methods described by AOAC procedures. Moisture content was determined by drying fish in oven at 103°C until a constant weight was achieved according to the AOAC (1990)procedures 925.40. Ash content was determined by incineration fish at 550°C according to the AOAC (1990) procedures 942.05. Nitrogen (N) content was determined using micro‐Kjeldahl method, according to AOAC (1990) procedures 984.13, the protein content was calculated as N × 6.25. Fat content was determined using Soxhlet apparatus with hexane, according to the AOAC (1990) procedures 963.15.

#### 2.4.2 Fatty acid composition of raw fish oil

Oils extracted from raw fish were use for the determination of fatty acid profile. Fatty acids composition of the fish oil were determinate after conversion of their fatty acid methyl esters (FAME) using borong trifluoride methanol method. The lipids were saponified and esterified for fatty acid analysis by the method described by Metcalfe, Schmitz, and Pelka (1966). The GC‐FID analyses were performed with an agilent (Agilent Technologies, Palo Alto, CA) 7890A series gas chromatograph equipped with FID detector using a DB‐225 capillary column (30 m × 0.25 mm, 0.25 μm of film thickness). The column temperature was initially maintained at 160°C for 2 min, increased to 220°C at 5°C/min and maintained for 10 min at 220°C. The carrier gas was nitrogen at a flow rate of 1.5 ml. The injector and detector temperatures were maintained at 230 and 250°C, respectively, with a split ratio of 50:1. Identification and quantification of fatty acids were based on comparison of their peaks with the relevant peak areas of the corresponding standard fatty acids where each fatty acid was then expressed as a percentage of the total fatty acids quantified.

### 2.5 Chemical analyses of fish oil

#### 2.5.1 Measurement of FFA of oil extracted from processing fish

The free fatty acids content was determined, according to AFNOR (1981) method, in the fat extracted by the Bligh and Dyer (1959). Sample (1 g) was dissolve in 100 ml of ethanol and 2 drops of phenolphthalein were added as an indicator and swirled vigorously. The mixture was titrated with potassium hydroxide (0.1 N). The free fatty acid (FFA) was expressed as % oleic acid.

#### 2.5.2 Analysis of IV of oil extracted from processing fish

The iodine value (IV) was determined using the Wijs method, as described by O'keefe and Pike (2010). The sample (0.2 g) of oil was dissolved in 15 ml of cyclohexane‐acetic acid (1:1) solvent. Then the mixture 25 ml of wijs solution was added, and the solution was kept in the dark at room temperature for 1 hr. Then, the mixture 20 ml of 15% KI solution and 150 ml of distilled water was added. The mixture was gradually titrated against 0.1 mol/L Na_2_S_2_O_3_ solution while continuously being vigorously shaken until the dark brown color disappeared. The blank was analyzed under the same conditions. The IV was expressed as g I_2_/100 g of samples.

#### 2.5.3 Measurement of PV of oil extracted from processing fish

The peroxide value (PV) was determined in the oil according to the method described by Low and Ng (1978), and the results were expressed in milliequivalents of O_2_/kg of lipid. Determinations were performed in triplicate.

#### 2.5.4 Fourier‐transform infrared spectra analysis

Infrared spectra between 4000 and 650 cm^−1^ were recorded using a perkin‐Elmer Spectrum 400 Infrared Spectrometer (Perkin‐Elmer Inc., Walthamn %A, USA) equipped with an ATR prism crystal accessory. The spectra resolution was 4 cm^−1^. Measurement were performed at room temperature using approximately 25 mg of the extracted oil, which were placed on the surface of the ATR crystal, and pressed with a flat‐tip plunger until spectra with suitable peaks were obtained. All experiments were performed in triplicate. Background was subtracted using the Spectrum software version 6.32 (Perkin‐Elmer Inc.).

### 2.6 Statistical analysis

The data were statistically analyzed using the Microsoft Excell 2010 software for Windows. Results were expressed as mean ± S.D. Results have been submitted to the analysis of variance (ANOVA) at 0.05% probability level. The Bonferroni test was used to compare means using the software Graphpad‐Instat, 2000.

## 3 RESULT AND DISCUSSION

### 3.1 Proximate composition of raw herring

Mean values of percentage of moisture, fat, protein, and ash of raw herring as determined by standard method are presented in Table 1. The moisture content in raw herring was 78.97%. Compared to raw mackerel (73%, Nazemrouya, Sahari, & Rezaei, 2011), this value is lower. The average lipid content obtained in this study was higher than 1.52%, the value reported for kukum caught from a Dam Lake in Turkey (Ozogul, Ozogul, & Alagoz, 2007), but rather low than the value reported for a raw sardine (Garcia‐Arias, Alvarez Pontes, Garcia‐Linares, Garcia‐Fernandez, & Sanchez‐Muniz, 2003). The variation in the lipid content might be due by some biological and environmental factors such as specie, food, temperature, location (Alvarez et al., 2009). Based on the classification of Suriah, Huah, & Duad, 1995; herring may be classified as fat fish because had lipid content higher than 10%. The crude protein content of herring was 69.43%. This value was higher than those found for mackerel fish (*Scomberomorus guttatus*) (16%) (Alvarez et al., 2009), catfish (*Arius maculatus*) (64%) (Tenyang et al., 2013). The ash content was 19.42% and can be positively correlated with mineral content in herring. This content was higher than that in three freshwater fish species in Pakistan (7‐12%) (Farhat & Abdul, 2011).

**Table 1:** Proximate composition of herring (*Clupea harengus*), g/100 g dry weight

Components	Composition (means ± *SD*)
Moisture	78.97 ± 0.32
Fat	10.20 ± 1.12
Proteins	69.43 ± 0.72
Ash	19.42 ± 0.09

*SD*, standard deviation, *n* = 3.

### 3.2 Fatty acid composition of raw herring

Table 2 shows the main fatty acids detected in the raw herring fish oil. Raw herring muscle lipid contained 40.29% of saturated fatty acids (SFA), 35.61% of monounsaturated fatty acids (MUFA), and 20.19% of polyunsaturated total fatty acids (PUFA). Among SFA, palmitic acid (C16:0) was the most important, followed by myristic acid (C14:0) fatty acid and stearic acid (C18:0). Lauric acid (C12:0) was the least SFA. Concerning the MUFA, oleic acid (C18:1) was the most important, followed by palmitoic acid (C16:1). Some reports indicated the similar results concerning the percentage of palmitic and oleic acid obtained in this study (Musaiger & D'souza, 2011). The value of palmitoic acid (8.22%) reported in this work was lower than 11% obtained to the diamond mullet in Bahrain (Musaiger & D'souza, 2011). This acid does not seem to be characteristic for any particular fish oil. The total amount of PUFA, including omega‐3, omega‐6, and others fatty acids. The presence of these two groups of PUFAs in diets are important for health because contribute to the reduction in incidence of cardiovascular diseases (Schram et al., 2007). The *n*‐3 PUFA present in herring oil extract was higher compared to *n*‐6 PUFA. These observations are in accordance to the findings by Tenyang et al. (2013) for raw catfish. They are also in agreement with the funding of Abdulrahman and Reshma (2011), who reported the same trend in fish consumed in Bahrain. The important omega‐3 PUFA such as EPA and DHA present in herring oil are very important because may be effective in epilepsy, cardiovascular diseases, arthritis, and mood stabilizers for bipolar disorder (Breslow, 2006). Linoleic acid (C18:2n‐6) was the most representative omega‐6 fatty acid family, followed by arachidonic acid (C20:4n‐6). Arachidonic acid contained in herring oil is a precursor of certain bio molecules such as prostaglandin and thromboxane, which interfere the blood clotting process (Abd Rahman, The, Osman, & Daud, 1995). The difference between the fatty acid compositions obtained in this study compared to another works could be attributed to the seasonal variation, the location, or and the diet. Aidos, Vander Padt, Luten, and Boom (2002) in their study demonstrated that the composition of fatty acids varied with seasonal changes.

**Table 2:** Fatty acid composition (% of total fatty acid) of raw herring

Fatty acid	% of total fatty acid
C12:0	0.20 ± 0.01
C14:0	6.22 ± 0.07
C16:0	28.79 ± 0.01
C18:0	5.08 ± 0.01
C20:0	nd
C22:0	nd
ΣAGS	40.29 ± 0.10
C16:1	8.22 ± 0.12
C18:1n9	27.39 ± 0.21
C20:1	nd
C22:1	nd
ΣAGMI	35.61 ± 0.33
C18:2n6	4.89 ± 0.09
C18:3n6	0.97 ± 0.00
C18:3n3	0.63 ± 0.01
C20:4n6	1.29 ± 0.01
C20:5n3 (EPA)	5.75 ± 0.04
C22:4n6	0.67 ± 0.07
C22:5n3	1.63 ± 0.06
C22:6n3 (DHA)	4.36 ± 0.02
ΣAGPI	20.19 ± 0.30
NI	4.33
Σn‐3	12.37
Σn‐6	7.82
*n*‐3/*n*‐6	1.58
AGPI/AGS	0.50

Values are means ± *SD* (*n* = 3); nd, nondetected. NI, nonidentified.

Table 2 also shows the ratios of PUFA/SFA and ω‐3/ω‐6 in oil extracted in raw herring. The PUFA/SFA ratio obtained revealed that raw herring oil is a good source of PUFA. That ratio was less than 1 and more than 0.45, minimum value recommended. According to Wood et al. (Wood et al., 2003), the risk of cancer and coronary heart disease is reduced where PUFA/SFA in diet is greater than 0.4. ω‐3/ω‐6 ratio of 1:1 or 1.1.5 can also contribute to the healthy diet in humans. The results noted for raw herring was higher and similar to those reported by Kalyoncu, Yaman, and Aktumsek (2010) for rainbow trout from Ivriz Dam Lake in Turkey. This high value indicated that herring fish used in this study and commonly consumed in Cameroon is more nutritive.

### 3.3 Free fatty acids value (FFA) of oils extracted from processed herring

#### 3.3.1 Smoking treatment

The changes in FFA of herring during traditional smoking process are shown in Table 3. The FFAs were used to determine the degree of lipolisis in processing herring. Lipid hydrolysis is one of the important changes that occur in fat fish muscle during post morten and during treatment with release of FFAs. On the one hand, FFAs formation during a relatively short chilled time occurs due to catalysis by endogenous enzymes, and only microbial effects would be significant after the end of the lag phase. Furthermore, during a thermal treatment, breakdown of triglycerides and phospholipids would be likely to occur and be the source of new FFAs formation (Whitte, Hardy, & Hobbs, 1990). The FFA of initial raw herring oil was 3.73% oleic acid. After hot smoking of unbleached herring, the value significantly (*p *˂ .05) increased. It was noted that the FFA of bleaching hot smoking herring was not significantly (*p* > .05) affected. Bleached smoked herring compared to unbleached smoked herring has the lower FFA. This may be explain by the inactivation of endogenous hydrolytic enzymes during the bleaching process. Such increases of FFAs during hot smoking of unbleached herring are due essentially of thermal lipolisis. These agree with the results obtained by Tenyang et al. (2013) when study the effect of traditional smoking on catfish.

**Table 3:** Changes in acid, iodine, and PV of herring oils during processing

	Samples	Acidity (% oleic acid)	Iodine value (g I_2_/100 g of oil)	Peroxide value (meq O_2_/kg of oil)
Smoking treatment	Raw	3.73 ± 0.34^b^	88.54 ± 1.34^a^	23.31 ± 1.26^b^
	Hot smoked	11.09 ± 0.27^a^	78.65 ± 0.93^b^	30.12 ± 0.85^a^
	Bleached + hot smoked	4.06 ± 0.38^b^	76.14 ± 3.36^b^	25.65 ± 1.08^b^
Refrigeration treatment	Raw (0 day)	3.73 ± 0.34^c^	88.54 ± 1.34^a^	23.31 ± 1.26^c^
	3 days	4.66 ± 0.83^c^	68.73 ± 0.00^b^	25.02 ± 0.47^c^
	6 days	9.94 ± 0.08^b^	62.51 ± 0.69^b^	34.50 ± 1.07^a^
	9 days	12.89 ± 0.36^a^	53.52 ± 3.01^c^	28.01 ± 0.86^b^

Values are means ± *SD* (*n* = 3). Mean values in the same column with different superscript letters are significantly different (*p* < .05).

The formation of FFA itself does not lead to nutritional losses. However, the accumulation of FFA has been related to some extend to lack of acceptability, because FFA oxidize faster than triglycerides and phospholipids.

#### 3.3.2 Refrigeration treatment

The Table 3 delineates the FFA value of the fish lipid extracted from the muscle of herring refrigerated at the day 0, 3, 6, and 9. In this study, no significant (*p* > .05) changes in FFA content were detected during the first 3 days of refrigeration at 4 °C. However, significant modifications were observed from day 6 and herring refrigerated at 9 days has the highest value of FFAs. Ours results are in agreement with those obtained by Nusrat, Fyrah, Sherazi, and Bhanger (2010), who noted the increase in FFAs content in freshwater Jarko fish during refrigeration. Increase in FFAs content with increase in time of refrigeration is generally associated to lipase activity originating from some microorganisms or some biological tissue. It is important to note that the fish used in this study neither sterilized, thus it is possible that some enzyme or micro organism contamination might have taken place during sample removal, which is contrast to earlier studies which reported that lipid hydrolysis of fish was greatly reduced upon sterilization (Dekoning, 1999). As per quality specifications for crude fish oil, maximum acceptable values of 5% FFA were proposed (Bimbo, 1998). The results obtained in this study showed that the FFA content of herring oil reached 5% limit during less than 6 days of refrigerated storage.

### 3.4 Iodine value (IV) of oils extracted from processed herring

#### 3.4.1 Smoking treatment

Raw herring contained more 50% of unsaturated fatty acid. These fatty acids are susceptible to lipid oxidation. During the smoking process, the double bonds of the fatty acids are attacked by free radicals, which results in the formation of conjugated bonds. Besides, some of the doubles bonds were destroyed during auto oxidation (Zhang et al., 2010). Thus, measuring the amount of unsaturated fatty acids present in fish oil can be used as reference to determine the quality of fish oil. The IV of oil extracted from raw and smoked herring fish are presented in Table 3. Initial IV of raw herring oil was 88.54 g I_2_/100 g of oil, but decrease significantly after hot smoking. The similar results were noted by Tenyang et al. (2013), during the analysis of effect of smoking on lipid oxidation of catfish. Generally, decrease in IV indicates the decrease in total unsaturated fatty acid during treatment. The decrease in IV during smoking could be attributed to the changes in fatty acids taking place with temperature and time of smoking.

### 3.5 Refrigeration treatment

The results presented in Table 3, show the effects of refrigeration time on iodine value of herring fish. A progressive decrease in IV was observed during refrigeration and refrigerated herring for 9 day has the lowest IV (53.52 g I_2_/100 g of oil). The rate of decrease in IV coincides with the rate of production of free fatty acids presented Table 3. The progressive decrease in IV observed in herring fish during refrigeration may be due to the action of oxidative enzymes which facilitate the reaction of oxygen to the double bond of unsaturated free fatty acid produced by lipolytic enzyme. These enzymes remained active at low temperature (Cho et al., 1989). Our current results are consistent with the research on Oily Monterey sardine stored at 0 °C during 15 days, which suggest that refrigeration duration increases the lipid oxidation of fish. The high iodine value in raw herring suggested that the herring oil is a source of unsaturated fatty acid that possess health benefits, such as regulating blood cholesterol levels and lowering elevated blood pressure (Gladyshev, Sushchik, Gubanenko, Demirchieva, and Kalachova). In contrast, refrigerated herring at 9 days had the lowest value of iodine value.

#### 3.5.1 PV of oils extracted from processed herring Smoking treatment

An important stage in the oxidation is the reaction of oxygen with the unsaturated fatty acid molecules to form hydroperoxide. The amount of these can be used as a measure of the extent of oxidation in the early stage. The peroxide test is a measure of the formation of hydroperoxides. The PV of raw and smoked herring is expressed in Table 3. The initial PV of raw herring was 23.31 meq O_2_/kg of oil. The PV of all smoked herring increased significantly (*p* < .05) throughout processing and unbleached smoked herring compared to bleached smoked herring had the higher PV (30.12 meq O_2_/kg of oil). The increases of PV in smoked herring are in agreement with the finding of Adeyeye, Oyewole, Obadina, and Omemu (2015) when study the effect smoking process on lipid quality of Bonga shad (*Ethmalosa frimbriata*) fish from Lagos State in Nigeria. These finding are also in accordance with the results obtained by Beltran and Moral (1989) on hot smoking sardine fillets, which noted the oxidation of PUFAs present in fish oil. Heating disrupt herring tissues, thereby facilitating contact between the unsaturated fatty acid rich in herring fish and pro‐oxidant in cells. The hydroperoxide form remained the primary oxidation products catalyzed by heat and light (Bremner, 2002). Bimbo (1998) suggested that the rancidity flavor occur when PV reach to 20‐40 meq O_2_/kg of oil. Therefore, the present investigation found that all smoked fish have showed rancidity flavor.

#### 3.5.2 Refrigeration treatment

Damage on unsaturated fatty acids during refrigeration process was also measured by PV. As can be seen from Table 3, the PV of refrigerated herring oil had changed compared to the PV of the oil from raw herring. Marked increase in PV was observed in herring muscle throughout the refrigeration process up to 6 days (*p *˂ .05). Thereafter, a decrease in PV was noticeable at day 9 of storage (*p *˂ .05). The increases in PV coincides with the rate of production of free fatty acids presented in Table 3. The change in PV is due to the increase in oxidation of fish lipid rich in unsaturated fatty acid caused by oxidative enzymes and pro‐oxidants contained in fish muscle, which accelerates the oxidation process and increases the rate of rancidity. Hydroperoxide formed during oxidation were rapidly break down in several steps, yielding a wide variety of decomposition products, including aldehydes, ketones, and epoxide (Nawar, 1996), because they are frequently unstable. This explains the reduction in PV obtained at day 9 of refrigeration. According to Manat, Soottawat, Wonnop, and Cameron (2006), ice storage increases significantly the oxidation of sardine (*Sardinella gibbosa*). Oxidation of fish lipid not only produces rancid odors and flavors, but also can be decrease nutritional quality and safety by the formation of primary oxidations products. These products are rapidly transformed in secondary product. The lipid oxidation products are known to be health hazards since they are associated with aging, membrane damage, heart disease, and cancer (Niki, 2009).

### 3.6 Change in FTIR spectra

#### 3.6.1 Smoking treatment

**Figure 1:**
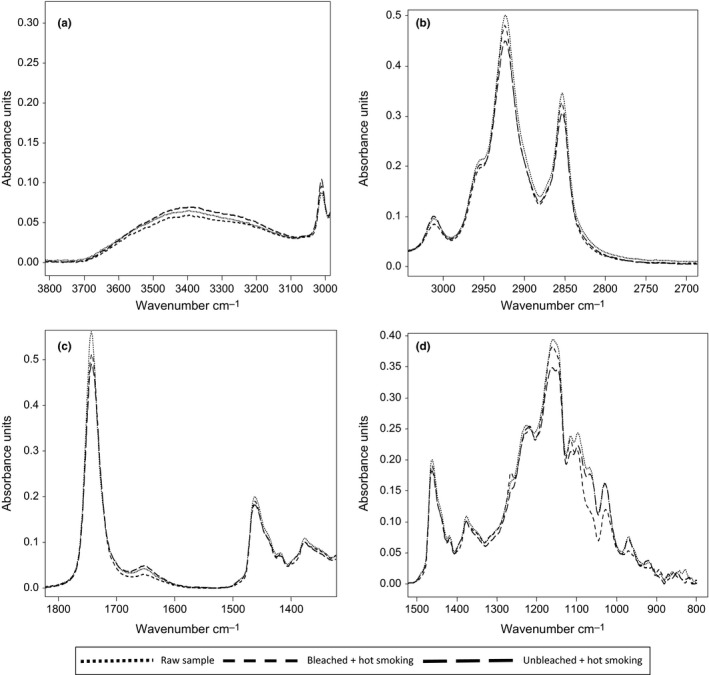
Selected regions (a–d) of Fourier transform infrared spectra of lipid extracted from smoked herring

Changes in Fourier‐transform infrared (FTIR) spectra of crude oil extracted from herring muscle during smoking are depicted in Figure 1. As seen from the figure, the spectrum of herring sample is quite complex and contains several bands arising from the contribution of different functional groups belonging to lipids and others. The characteristic absorption peaks of herring oil appear at three distinct frequency ranges, namely 3700–3050 cm^−1^, 3050–2800 cm^−1^, 1800–1400 cm^−1^, and 1500–800 cm^−1^ region. FTIR spectra of raw and treated herring oils shows that there exist notable difference in the band around 3350 cm^−1^ (Figure 1a) assigned to the OH stretching vibration of hydroperoxides. Unbleached smoked herring presented a high absorbance. These results were coincidental with those obtained by the titrimetric methods (Table 3). The analytical signal used to determine unsaturation was the peak at 3012 cm^−1^ (Figure 1a), due to the stretching vibration of the C‐H link adjoining the double C = C link. The result obtained show how the intensity of raw and unbleached smoked herring had sensibly the same and high, compared to bleached sample. These could be due to the higher unsaturated fatty acid contained in these samples. Absorption bands at 2925 cm^−1^ and 2850 cm^−1^ (Figure 1b) correspond to asymmetric and symmetric stretching vibrations of methyl (CH_3_) groups, respectively. The former band mainly monitors the lipids and the latter mainly monitors the proteins in the biological system (Korkmaz & Severcan, 2005). As can be seen from Figure 1b, there are most variation in the bands at 2925 and 2850 cm^−1^ and unbleached smoked herring presented the less absorbance band. These finding indicate an appreciable lowering of the concentration of CH_3_ and CH_2_ functional groups in the treated samples. Vlachos et al. (2006) reported a reduction in the absorbance of these bands as related to oxidative changes in vegetable oils. The other frequency range under consideration was 1800–1500 cm^−1^ (Figure 1c). It is clearly seen from that region that the absorbance values of bands generally changed. The sharp and narrow band observed at 1750 cm^−1^ is assigned to C=O stretching vibration of ester groups in triacylglycerols (Vlachos et al., 2006). A decrease in the absorbance at this wave number was visible after smoking and the lowest band is related to unbleached hot smoked herring (Figure 1c). A decrease in the intensity of the lipid C=O stretching vibration at 1750 cm^−1^ suggested a decreased concentration of the ester group belonging of triglyceride within the smoked fish (Cakmak, Togan, Uduz, & Severcan, 2003). According to Guillén, Ruiz, and Cabo (2004), these changes are produces by the appearance of aldehydes and ketones, which are secondary oxidation products, derive from the degradation of hydroperoxides. Two bands at 1461 cm^−1^ and 1375 cm^−1^ (Figure 1d) are due to CH absorption bending vibration of CH_2_ and CH_3_ groups, respectively (Vlachos et al., 2006). The bands near 1250 and 1160 cm^−1^ associate with the stretching vibration of the C‐O ester groups and with bending vibration of change during oxidation treatment. Sharp decrease in absorbance value of the band near 1250 cm^−1^ refers to oxidation process. In this study, bleached smoked herring had the lower absorbance. The absorbance at 1147 cm^−1^ showed a clear tendency to decrease and unbleached smoked sample presented the lower band. That peak is related to the proportion of saturated acyl group. The absorbance of the band at 967 cm^−1^, associated with bending vibrations of CH functional group of isolated trans‐olefins, increases as oxidation advances. Bands of wave number of 973 and 976 cm^−1^ refers to the possible aldehydic and ketonic groups with isolated trans double bonds. Therefore, the higher absorbance in these wave number is the indication of the advanced oxidation process. In the case of our study, any changes in these wave number were not observed.

#### 3.6.2 Refrigeration treatment

**Figure 2:**
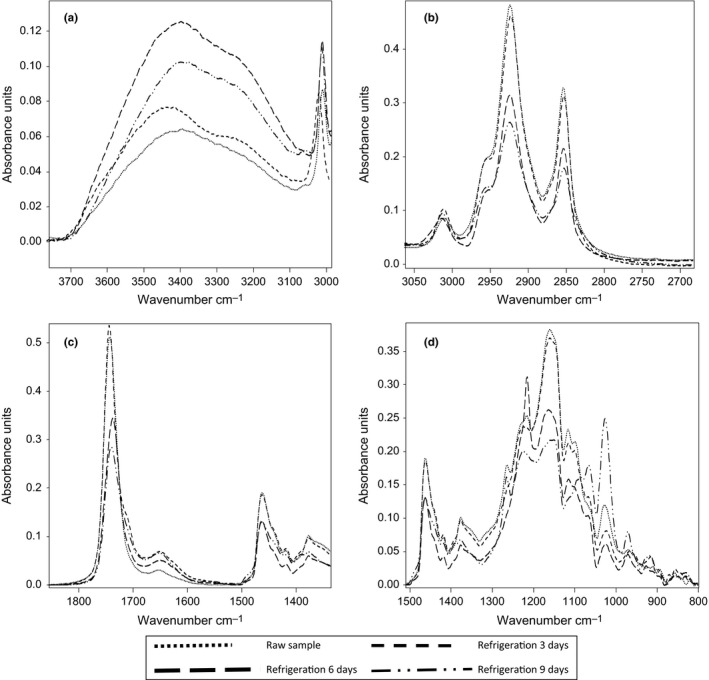
Selected regions (a–d) of Fourier transform infrared spectra of lipid extracted from refrigerated herring

The differences in the FTIR spectra, due to oxidation of herring during refrigeration storage at day 0, 3, 6, and 9 are presented in Figure 2. In the region of wave number 3800–3100 cm^−1^ (Figure 2a), stretching vibration of the OH of hydroperoxide form increase, and after the first 6 days, the absorbance band decrease. Refrigerated sample at 6 days had the higher absorbance band, whereas raw sample had the less absorbance. The decrease in the band at this wave number suggesting the decomposition of hydroperoxide to yield secondary lipid oxidation product. These observations are in agreement with those obtained by the titrimetric method. Van de Voort, Ismail, Sedman, Dubois, and Nicodemo (1994), reported absorbance at 3800–3100 cm^−1^ in the ATR/FTIR spectra, referred to as the OH stretching region. Hydroperoxide moieties exhibit characteristic absorption bands between 3600 and 3400 cm^−1^ due to their –OOH stretching vibration. It contrast to the findings reported in salmon lipids by Vlachos et al. (2006). A reduction in the degree of lipid unsaturation due to an eventual oxidative process during the refrigeration period could not be evidenced by monitoring the peak at 3012 cm^−1^ (Figure 2b). However, the bands at 2925 and 2850 cm^−1^ showed a reduced absorbance at days 6 and 9 of refrigeration. These finding indicated an appreciable lowering of the concentration of CH_3_ and CH_2_ functional groups in this sample. Vlachos et al. (2006) also reported a reduction in the absorbance of the bands as related to oxidative changes in vegetable oils. Changes in the carbonyl absorption of the triglycerides ester linkage at around 1750 cm^−1^ (Figure 2c), were reported as a main FTIR event denoting lipid oxidation. A decrease in the absorbance at this wave number was visible during refrigeration duration and refrigerated herring for 9 days had the lower absorbance (Figure 2c). The bands associated with the fingerprint region observed between 1500 and 1000 cm^−1^ (Figure 2d) were different throughout the refrigeration duration. The results obtained at 1460 and 1370 cm^−1^ give the similar conclusion with those obtained at 2925 and 2850 cm^−1^. The spectral region between 1280 and 1050 cm^−1^ was most altered at days 6 and 9 of refrigeration. The absorbance of the peaks at 1200 and 1025 cm^−1^ showed a clear tendency to decrease at day 6 and 9. Both peaks are related to the proportion of saturated acyl groups.

## 4 CONCLUSION

Accordingly, based on our results, we can say that herring fish analyzed is a good source of proteins, lipid, and ash. It also has valuable nutritional characteristics due to its high PUFAs content, especially EPA and DHA. The *n*‐3/*n*‐6 ratios are within the recommended values by some researchers, thus constituting a healthy food.

Processing (smoking, refrigeration) generally had the most deleterious effect on lipid oxidation of herring oil. The change in the proportion of FFAs, IV, PV, and triglycerides revealed a high susceptibity to these treatments on lipid oxidation of herring.

The results also suggested that the most changes in proportion of FFA, IV, PV, and triglycerides of herring occurred at most of 6 days of refrigeration and during unbleaching smoking treatment. This species of fish, if immediately refrigerated can be usable up to less than 5 days. Bleaching combined to smoking was found to be the best method to smoke herring.

## CONFLICT OF INTEREST

None declared.
